# Altered endoplasmic reticulum calcium loading in human PLN-R14del cardiomyopathy

**DOI:** 10.3389/fcell.2025.1627985

**Published:** 2025-07-28

**Authors:** Willem Borbein, Lukas Dahmlos, Umber Saleem, Marina Reinsch, Ingke Braren, Thomas Schulze, Birgit Klampe, Friederike Cuello, Justus Stenzig, Thomas Eschenhagen, Arne Hansen

**Affiliations:** ^1^ Department of Experimental Pharmacology and Toxicology, University Medical Center Hamburg-Eppendorf, Hamburg, Germany; ^2^ German Center for Cardiovascular Research (DZHK), Partner site Hamburg/Lübeck/Kiel, Berlin, Germany; ^3^ Vector Core Unit, University Medical Center Hamburg-Eppendorf, Hamburg, Germany

**Keywords:** phospholamban R14del, human induced pluripotent stem cells, engineered heart tissue, endoplasmic reticulum, CEPIAer

## Abstract

The phospholamban (PLN) R14del genetic variant causes dilated cardiomyopathy. Previous studies suggest involvement of the ER stress response and impairment of ER-related signaling pathways such as autophagy. In this study, human-induced pluripotent stem cell (hiPSC)-derived cardiomyocytes (CMs) from an unrelated control subject, a PLN-R14del patient, and a corresponding isogenic control were transduced with adeno-associated virus serotype 6 (AAV6) encoding the endoplasmic reticulum calcium sensor CEPIAer. Indicator compounds for the modulation of ER calcium homeostasis showed similar characteristic effects on CEPIAer fluorescence intensity in engineered heart tissues (EHTs) from all three cell lines, validating CEPIAer fluorescence intensity as a surrogate for ER calcium loading. Cytoplasmic calcium loading induced by high extracellular calcium concentration revealed subtle alterations in PLN-R14del that were consistent with higher ER calcium loading. FACS analyses of dissociated cardiomyocytes confirmed higher ER calcium load. Taken together, this study provides evidence for altered ER calcium loading as a new disease mechanism in PLN-R14del cardiomyopathy.

## Introduction

Phospholamban (PLN) localizes to the membrane of the sarcoplasmic and endoplasmic reticulum (SR and ER) and regulates sarco/endoplasmic reticulum Ca^2+^-ATPase (SERCA2)-mediated Ca^2+^ uptake. In human cardiomyocytes (CMs), excitation–contraction coupling (EC coupling) is based on a finely tuned interaction between the SR and the Na^+^/Ca^2+^ exchanger (NCX). The SR serves as a primary intracellular Ca^2+^ store that releases Ca^2+^ via ryanodine receptors (RyRs) upon the activation of the L-type Ca^2+^ channel, a process known as Ca^2+^-induced Ca^2+^ release (CICR). SERCA2a mediates Ca^2+^-reuptake into the SR, while NCX facilitates Ca^2+^-extrusion via cellular membranes (Aronsen et al., 2013; Bers, 2002). Ca^2+^ uptake into the ER is important to maintain high luminal Ca^2+^ concentrations and functions (Eijgenraam et al., 2020; Eijgenraam et al., 2021).

The PLN-R14del genetic variant is associated with dilated cardiomyopathy in patients. Penetrance of the PLN-R14del variant is age-related and up to 70% (Verstraelen et al., 2025). Several PLN-R14del mouse models revealed an impact on Ca^2+^ handling and SERCA2a regulation. Early transgenic models overexpressing the PLN-R14del protein indicated SERCA super-inhibition and impaired Ca^2+^ reuptake (Haghighi et al., 2006), but this effect was not confirmed in a PLN-R14del model without the presence of the wild-type protein (Haghighi et al., 2012). Knock-in mice expressing a murine PLN-R14del variant showed a loss of SERCA inhibition, not enhancement, with a dose-dependent effect (Stege et al., 2023; Maniezzi et al., 2024). These findings challenge the idea of SERCA2 super-inhibition in PLN-R14del cardiomyopathy and suggest a loss-of-function effect.

Additional studies in mice, human failing heart samples, and human-induced pluripotent stem cell (hiPSC) models suggest the involvement of ER stress and related pathways such as autophagy (Eijgenraam et al., 2020; Eijgenraam et al., 2021; Te Rijdt et al., 2016; Te Rijdt et al., 2017; Vafiadaki et al., 2024; Cuello et al., 2021; Feyen et al., 2021). Homozygous PLN-R14del knock-in mice showed a strong cardiomyopathy phenotype, accompanied by histological evidence of PLN aggregate formation. Aggregate appearance preceded the functional deficit and was associated with disruptions in protein homeostasis pathways (Eijgenraam et al., 2020; Eijgenraam et al., 2021). Studies in human failing heart samples from PLN-R14del patients revealed perinuclear PLN aggregate formation in cardiomyocytes and their co-localization with markers for autophagy (Te Rijdt et al., 2016; Te Rijdt et al., 2017). PLN aggregates were identified as autophagosomes that were unable to fuse with lysosomes, resulting in diminished autophagic flux (Vafiadaki et al., 2024). Patient-derived PLN-R14del hiPSC models demonstrated an ER disease phenotype without the overt alteration of sarcoplasmic reticulum function (Cuello et al., 2021; Feyen et al., 2021). Although a higher frequency of cytoplasmic Ca^2+^ irregularities and altered Ca^2+^ kinetics were reported as indicative of SR changes, these alterations do not appear to compromise force regulation (Cuello et al., 2021; Badone et al., 2021).

ER structures have been identified in cardiomyocytes in the peri-nuclear region and near the Z-lines (Michalak and Opas, 2009). Important ER functions include protein biosynthesis and post-translational modification, protein folding and degradation of misfolded proteins by the proteasome, so-called ER-associated degradation (ERAD), lipid and steroid metabolism, and drug detoxification. The total luminal ER Ca^2+^ concentration is in the range of up to 800 μM, and the free luminal Ca^2+^ concentration is 100–200 µM. The remaining Ca^2+^ is bound to ER-resident proteins such as calreticulin, immunoglobulin-binding protein (BiP), glucose-regulated protein 94 (GRP94), and protein disulfide isomerase (PDI), which serve as Ca^2+^ buffers and are also involved in biological functions (Samtleben et al., 2013; Prins and Michalak, 2011). The cellular volume fractions of the ER and SR are similar (Page and McCallister, 1973), pointing to the importance of the ER Ca^2+^ content for cardiomyocyte Ca^2+^ homeostasis. Important mechanisms for the uptake of Ca^2+^ into the ER are the plasma membrane-activated Ca^2+^ release-activated calcium channels (CRACs) from the extracellular compartment (Orai/STIM, stromal interaction molecule) (Derler et al., 2016; Liou et al., 2005) and SERCA2 from the cytoplasm (Reddish et al., 2021). Ca^2+^ is released from the ER via inositol 1,4,5-trisphosphate receptors (IP_3_Rs) and RyRs (Suzuki et al., 2014). Dysregulation of Ca^2+^ homeostasis leads to ER dysfunction and stress responses (Daverkausen-Fischer and Pröls, 2022; Groenendyk et al., 2021). The ER–mitochondrial interface describes a contact site between the two organelles that mediates Ca^2+^ flow from the ER to the mitochondria, thereby stimulating mitochondrial oxidative metabolism and replication. This tightly controlled Ca^2+^ transfer is mediated by IP_3_Rs, voltage-dependent anion channels (VDACs), and the chaperone glucose-regulated protein 75 (GRP75) (de Ridder et al., 2023; Lewis et al., 2016). The genetically encoded ER Ca^2+^ sensor CEPIAer was developed to study ER Ca^2+^ homeostasis. CEPIAer is equipped with an ER targeting and retention sequence to ensure specific subcellular targeting to the ER and enable the investigation of CEPIAer fluorescence intensity (FI) as a surrogate for ER Ca^2+^ loading (Suzuki et al., 2014).

In this study, we establish the CEPIAer sensor in hiPSC-derived engineered heart tissues (EHTs). We show perinuclear CEPIAer localization in hiPSC-CMs. Indicator compounds with defined effects on ER Ca^2+^ show similar characteristic effects on CEPIAer FI in EHTs from the control, isogenic control (PLNic), and PLN-R14del hiPSC-CMs. Pharmacological modulation of SERCA2-mediated ER Ca^2+^ uptake unmasked subtle alterations in PLN-R14del ER Ca^2+^ homeostasis. FACS analysis of dissociated hiPSC-CMs from PLN-R14del confirms higher CEPIAer FI, indicating higher ER Ca^2+^ loading.

## Materials and methods

### HiPSC expansion and cardiac differentiation

An established hiPSC control line (hiPSCreg code: UKEi001-A, ERC01) and patient-derived PLN-R14del hiPSCs, along with the respective isogenic control (Cuello et al., 2021), were differentiated into cardiomyocytes. In brief, hiPSCs from the master/working cell bank (Shibamiya et al., 2020) were expanded on Geltrex-coated cell culture vessels in FTDA media (Frank et al., 2012). Embryoid bodies were generated in spinner flasks. Ventricular cardiomyocytes were differentiated in suspension/EB format by growth factor/small molecule cocktails into mesodermal progenitors and subsequently into cardiomyocytes. Collagenase-dissociated hiPSC-CMs were either cryo-preserved or directly used for EHT generation. Differentiation efficiency was determined by FACS analysis for troponin T. All procedures were performed as described by Breckwoldt et al. (2017).

### rAAV vector particle production and purification

The production and purification of adeno-associated virus serotype 6 (AAV6) vector particles encoding the calcium sensor G-CEPIA1er were adapted from a previous publication (Saleem et al., 2020). Recombinant adeno-associated virus serotype 6 (rAAV6) particles were produced using the baculovirus expression system techniques. To introduce the calcium sensor into the baculoviral transfer plasmid pFBGR-Ultra, a PCR fragment encoding G-CEPIAer, flanked by ER signaling and retention sequences (Suzuki et al., 2014), was generated using primers (5′-AACATCGATTGAATTCgccaccatgggatggagc and 5′- tatagggcgaattgggtaccctacagctcgtccttctcgct), with pCMV G-CEPIA1er (Addgene #58215) as a template. The fragment was inserted into pFBGR-Ultra TnT-GFP, previously digested with EcoRI and KpnI, using In-Fusion Cloning (PrimeSTAR GXL Polymerase, TaKaRa Bio Europe SAS, Cat. No. R050B). Final baculovirus transfer plasmids were confirmed by restriction digesting and whole plasmid sequencing (MWG Eurofins). The genomic titers of DNAse-resistant recombinant AAV6 particles were determined by quantitative PCR using T7/SV40 specific primers (5′-cctatagtgagtcgtattacgcgc and 5′-gctgcaataaacaagttgggccat).

### HiPSC-CM 2D culture and immunofluorescence

Control hiPSC-CMs were plated on Geltrex-coated 96-well plates in EHT media. HiPSC-CMs were incubated for 14–21 days, with media being changed every 2–3 days. For immunofluorescent staining, the media was removed. HiPSC-CMs were briefly washed with PBS and fixed with 4% paraformaldehyde for 15 min at 4 °C. Then, fixative was removed, and hiPSC-CMs were permeabilized with either 0.2% Triton X-100 (Roth 3051.3) or 0.2% saponin (Sigma, S7900, from Quillaja bark) in PBS for 5 min at room temperature (RT), followed by washing in 200 µL PBS/well for 5 min. Subsequently, non-specific binding sites were blocked with 5% normal goat serum (NGS) diluted in antibody buffer (10 mM Tris, 155 mM NaCl, 2 mM EGTA, 2 mM MgCl_2_, and 1% (w/v) BSA; pH 7.5) for 20 min at RT. For primary antibody incubation, cells were incubated overnight with 50 µL/well of the primary antibodies against calnexin (Novus, AF18), anti-alpha-actinin (Sigma-Aldrich, A7811), or SERCA2 (Invitrogen, MA3-919) diluted in antibody buffer (10 mM Tris, 155 mM NaCl, 2 mM EGTA, 2 mM MgCl_2_, and 1% (w/v) BSA; pH 7.5) at 4 °C in a humid chamber under shaking. After washing three times with 200 µL PBS/well, the cells were incubated with 50 µL of secondary antibody solution consisting of the secondary antibodies Alexa Fluor 647 goat anti-mouse (1:100, Invitrogen, A21236), DyLight 550 Goat Anti-Rabbit (1:100 DyLight 550, Abcam, ab96884), and 4′,6-diamidino-2-phenylindole (DAPI, 1:100, Sigma, D9542) in a humid chamber on a shaker for 3 h at RT.

### Generation of CEPIAer EHTs

Fibrin-based strip-format engineered heart tissues were generated with 1.0 × 10^6^ hiPSC-CMs per construct, as described by Breckwoldt et al. (2017). During EHT casting, hiPSC-CMs were transfected with AAV6-CEPIAer at a multiplicity of infection of 1.0 × 10^5^. EHTs were cultivated for approximately 21 days in EHT medium (10% horse serum, 1% penicillin–streptomycin, 0.1% aprotinin, 0.1% insulin, and 0.25% tranexamic acid diluted in Dulbecco’s modified Eagle medium) in 24-well plates with media changes on Mondays, Wednesdays, and Fridays.

### Functional assessment

The EHTs were transferred to a 24-well plate prefilled with EHT measurement medium (FluoroBrite DMEM, Thermo Fisher Scientific). The plate was transferred to an EHT contractility system with a customized extension. This extension consisted of an XYZ-axis controlled fluorescence detection unit (FDU, including an objective, a filter system, and a photomultiplier) mounted under the 24-well plate and integrated into the analysis software to enable simultaneous measurement of force and GFP fluorescence. XYZ-axis coordinates were defined for contour-based video-optical analysis of contractile force and fluorescence detection. Automated force/fluorescence analysis was performed at baseline and after the addition of pharmacological modulators of ER calcium loading and release. After the experiment was completed, the EHTs were washed three times in EHT medium (10% horse serum, 1% penicillin–streptomycin, 0.1% aprotinin, 0.1% insulin, and 0.25% tranexamic acid diluted in Dulbecco’s modified Eagle medium) and transferred back to the EHT media. All media and washing solutions were pre-equilibrated in a cell culture incubator. The pharmacological compounds used were as follows: isoprenaline (Sigma-Aldrich, I-5627), thapsigargin (Sigma-Aldrich, T9033), cyclopiazonic acid (Sigma-Aldrich, 239805), istaroxime (MedChemExpress, HY-15718A), ryanodine (Sigma-Aldrich, 559276), tetracaine (Sigma, T7508), and adenosine triphosphate (ATP) (Sigma, A6419). Thapsigargin-based *in vitro* assays have been established to induce ER calcium dysregulation and ER stress. The stimulation was carried out for up to 24 h (Abdullahi et al., 2017). In this study, the effects of thapsigargin and other indicator compounds were analyzed until the effects on CEPIAer FI reached plateau values and for a maximum period of up to 370 min.

### Quantitative PCR ER stress marker

EHTs were incubated in EHT measurement media (FluoroBrite DMEM, Thermo Fisher Scientific) in the presence of pharmacological modulators of ER calcium loading and release for the defined period of time. EHTs were briefly washed with pre-warmed (37 °C) PBS, transferred to 2.0-mL sample tubes, snap-frozen in liquid nitrogen, and stored at −80 °C. Total RNA was isolated using TRIzol reagent (Ambion, RNA by Life Technologies, 15596–026) according to the manufacturer’s instructions. RNA concentration and quality in the eluate were evaluated using NanoDrop instruments. Reverse transcription was performed using the High-Capacity cDNA Reverse Transcription Kit (Applied Biosystems 4368813) according to the manufacturer’s instructions. Quantitative real-time PCR was performed using 5x EvaGreen (Solis BioDyne) according to the manufacturer’s instructions. Primer sequences for ER stress response markers and housekeeping genes were used as described by Cuello et al. (2021).

### Flow cytometry

HiPSC-CM EHTs were dissociated using a 50% (v/v) papain solution (10 U/mL papain (Sigma-Aldrich, 76220), 1 mM EDTA (Roth 8043.2), and 5 mM L-cysteine-HCl (Sigma-Aldrich, C1276) in 1x EBSS (Gibco 14155–048)) diluted in HBSS (Gibco 14175–053). The EHTs were incubated for 30–40 min until single-cell dispersal was observed. Dissociated hiPSC-CMs were stored on ice in EHT media and later transferred to EHT measurement media. CEPIAer GFP FI was determined by FACSCanto II (BD). Analysis was performed using FACSDiva software (BD).

### Statistical analysis

Data were expressed as the mean ± SEM. GraphPad Prism was used to compare between groups with an unpaired, two-sided Student’s t test or one-way ANOVA, as indicated. Individual hiPSC-CM EHTs or single hiPSC-CMs from 2–3 different EHT generation batches (as indicated in the figure legends) were considered biological replicates.

## Results

### Subcellular localization of CEPIAer in hiPSC-CMs

**FIGURE 1:**
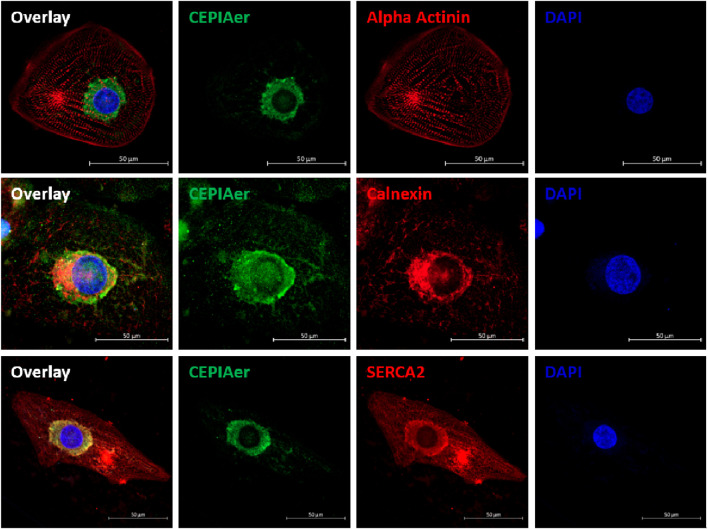
CEPIAer immunofluorescence. Immunofluorescence staining of 2D hiPSC-CMs after transduction with the CEPIAer sensor (green), staining using antibodies against alpha-actinin, the ER-resident protein calnexin, and SERCA2 (red, as indicated), along with DAPI staining (blue); scale bar 50 µm.

hiPSC-CMs were plated as a 2D monolayer and transduced with AAV6 encoding the CEPIAer Ca^2+^ sensor, flanked by endoplasmic reticulum targeting and retention sequences under the control of a troponin T promoter. This CEPIAer construct was previously characterized and shown to have ER-specific expression (Suzuki et al., 2014). Immunofluorescence staining revealed perinuclear CEPIAer fluorescence in alpha-actinin-positive hiPSC-CMs (Figure 1, upper panel). CEPIAer fluorescence co-localized with staining for the ER resident protein calnexin (Figure 1, middle panel) and with the perinuclear fraction of SERCA2 staining (Figure 1, lower panel).

### CEPIAer FI—baseline characterization

**FIGURE 2:**
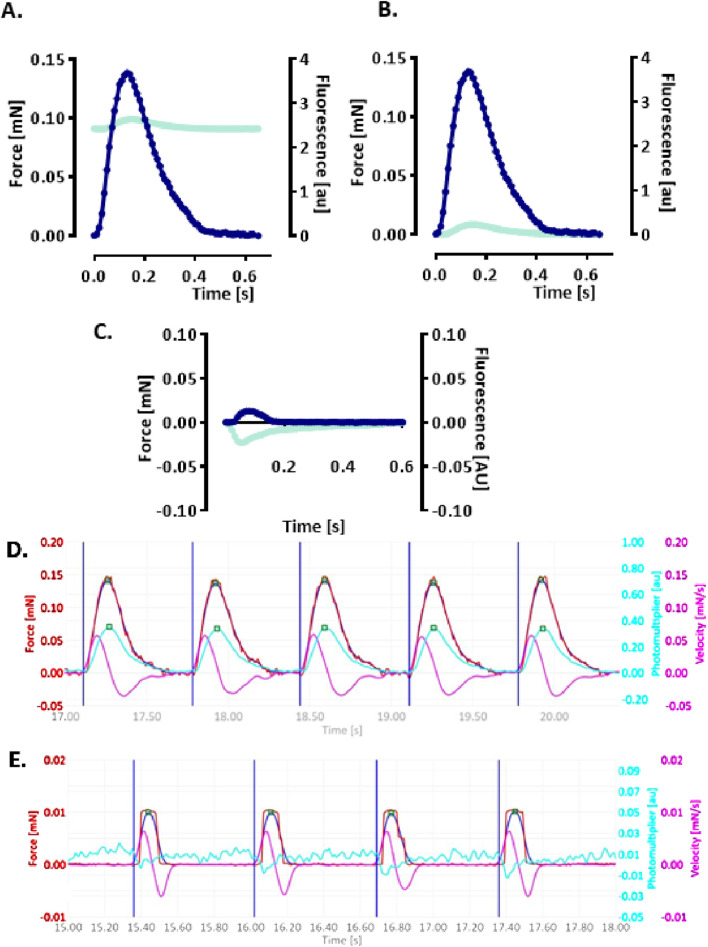
Simultaneous recording of CEPIAer fluorescence intensity and force **(A–C)**. **(A)** Average contraction (blue) and not baseline-corrected fluorescence intensity (cyan) of electrically stimulated EHTs from the control hiPSC line. **(B)** Same data plotted with baseline-corrected fluorescence intensity. **(C)** Average contraction (blue) and not baseline-corrected fluorescence intensity (cyan) of electrically stimulated control EHTs in the presence of myosin inhibitor butanedione monoxime (BDM, 10 mM). n = 7–8 EHTs per condition. Data are expressed as the mean. **(D)** Original baseline-corrected recording at baseline and **(E)** in the presence of BDM (10 mM). Vertical blue lines: electrical pacing signal; blue curve: non-filtered force curve; red line: Gaussian-filtered force curve; pink line: velocity of contraction/relaxation; cyan line: baseline-corrected CEPIAer fluorescence intensity.

A functional assay was established to simultaneously monitor CEPIAer FI and auxotonic contraction in spontaneously beating EHTs. Figure 2A shows the average contraction peak and non-baseline-corrected CEPIAer FI. The minor increase in FI during contraction is indicative of a motion artefact. The CEPIAer FI level of non-baseline-corrected recordings without taking the increase during contraction into account (e.g., 2.4 AU, Figure 2A) was quantified as a surrogate for ER Ca^2+^ loading in this study. The plot of average force and CEPIAer FI with baseline-correction is shown in Figure 2B, and an original recording is shown in Figure 2D. Pre-treatment with the myosin inhibitor butanedione monoxime (BDM, 10 mM), which suppresses mechanical shortening during electromechanical coupling, was performed in electrically paced EHTs. Here, force was reduced to the minimal values, and CEPIAer FI with baseline-correction did not show motion artifacts but instead showed a minimal decrease during contraction (Figures 2C, E).

### CEPIAer FI—effect of indicator compounds

**FIGURE 3:**
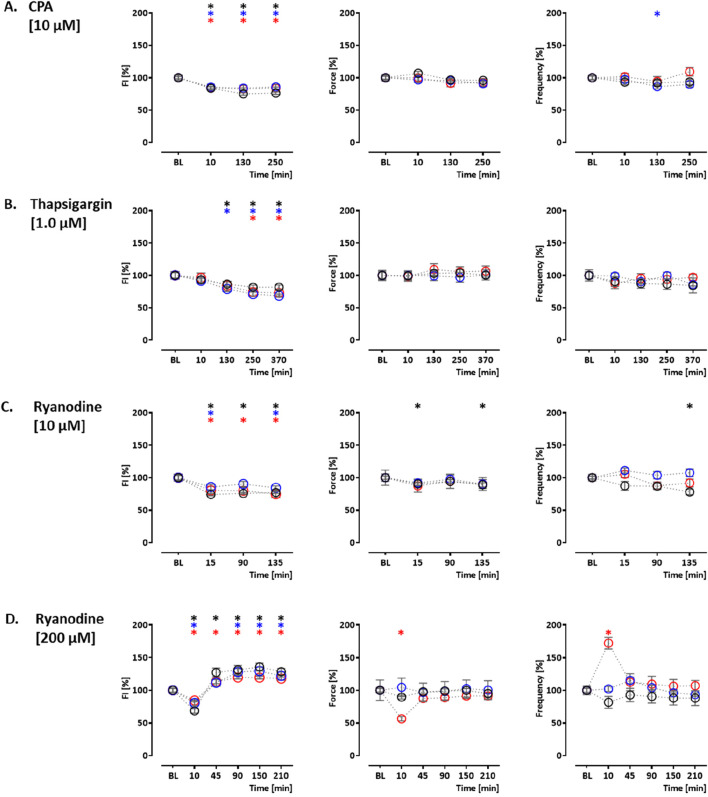
Drug response to cyclopiazonic acid, thapsigargin, and ryanodine. Effects on CEPIAer fluorescence intensity (left), force (middle), and frequency (right) of spontaneously beating hiPSC-CM EHTs from control (O), PLNic (O), and PLN-R14del hiPSC line (O). Data are plotted relative to mean baseline and normalized to time/vehicle control (TVC) per EHT batch. **(A)** Cyclopiazonic acid (10.0 µM; control: n = 15 EHT; TVC = 14 EHT; two batches; PLNic: n = 23 EHT; TVC = 23 EHT; three batches; PLN-R14del: n = 32 EHT; TVC = 22 EHT; three batches). **(B)** Thapsigargin (1.0 µM; control: n = 16 EHT; TVC = 14 EHT; two batches; PLNic: n = 16 EHT; TVC = 11 EHT; two batches; PLN-R14del: n = 19 EHT; TVC = 20 EHT; three batches). **(C)** Ryanodine (10.0 µM; control: n = 14 EHT; TVC = 13 EHT; two batches; PLNic: n = 25 EHT; TVC = 20 EHT; three batches; PLN-R14del: n = 21 EHT; TVC = 19 EHT; three batches). **(D)** Ryanodine (200.0 µM; control: n = 8 EHT; TVC = 8 EHT; two batches; PLNic: n = 7 EHT; TVC = 8 EHT; two batches; PLN-R14del: n = 8 EHT; TVC = 8 EHT; one batch). One-way ANOVA *versus* baseline with Dunnett’s post-test, *p < 0.05. Mean ± SEM. Asterisk color code indicates the significance for each hiPSC line. The scatter plots of the same data for the different cell lines are shown in Supplementary Figures S1, S2.

In order to validate the assay, the effects of indicator compounds on CEPIAer FI, force, and spontaneous beating frequency were analyzed. Time-dependent effects were studied in EHTs of a control hiPSC line (control) and the isogenic (PLNic) and PLN-R14del hiPSC line. This isogenic hiPSC pair was previously characterized and revealed cytoplasmic Ca^2+^ irregularities and lower force associated with the impairment of the ER/mitochondria compartment for PLN-R14del (Cuello et al., 2021). Cyclopiazonic acid and thapsigargin were studied as inhibitors of ER Ca^2+^ uptake. Cyclopiazonic acid is a competitive antagonist at the SERCA ATP-binding site (IC_50_, 0.6 µM (Seidler et al., 1989)). Thapsigargin is a non-competitive antagonist at the SERCA ATP-binding site (IC_50_, 1.0 nM (Lytton et al., 1991)). CPA (10 µM) and thapsigargin (1 µM) led to a time-dependent reduction in CEPIAer FI in all three cell lines (CPA: control: 76% ± 1.9%; PLNic: 86% ± 3.8%; and PLN-R14del: 84% ± 1.7% and thapsigargin: control: 82% ± 3.3%; PLNic: 69% ± 2.4%; and PLN-R14del: 73% ± 5.0%). No effect on force and frequency was detected. In PLNic, CPA led to a transient reduction in frequency (at 130 min, 86% ± 3.5%; Figures 3A, B, Supplementary Figures 1A, B).

**FIGURE 4:**
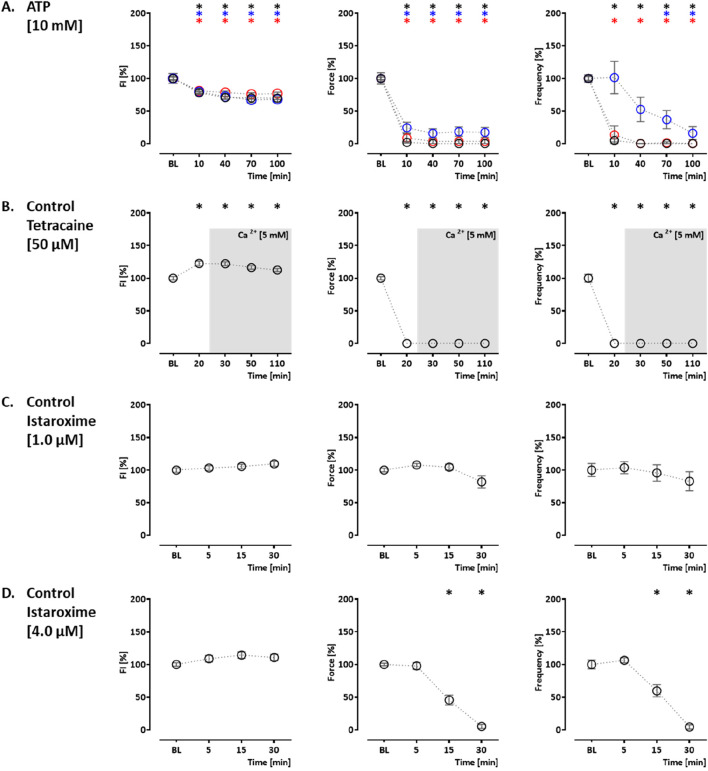
Drug response to ATP, tetracaine, and istaroxime. **(A)** Effects of ATP on CEPIAer fluorescence intensity (left), force (middle), and frequency (right) of spontaneously beating hiPSC-CM EHTs from control (O), PLNic (O), and PLN-R14del hiPSC line (O). Data are plotted relative to mean baseline and normalized to TVC per EHT batch. ATP (10.0 mM; n = 35 EHT; TVC = 30 EHT; four batches; PLNic: n = 16 EHT; TVC = 15 EHT; two batches; PLN-R14del: n = 25 EHT; TVC = 23 EHT; three batches). **(B)** Effects of tetracaine (50 µM) on hiPSC-CM EHTs from control hiPSC line (O). Tetracaine (50.0 µM; n = 20 EHT; TVC = 17 EHT; two batches)—for the tetracaine experiment, extracellular calcium concentration was increased to 5 mM after the 20-min time point, as indicated in the graphs. **(C, D)** Effects of istaroxime on hiPSC-CM EHTs from control hiPSC line (O). **(C)** Istaroxime (1.0 µM; control: n = 18 EHT; TVC = 17 EHT; three batches). **(D)** Istaroxime (4.0 µM; n = 28 EHT; TVC = 23 EHT; four batches. One-way ANOVA *versus* baseline with Dunnett’s post-test, *p < 0.05. Mean ± SEM. Asterisk color code indicates the significance for each hiPSC line. The scatter plots of the same data for the different cell lines are shown in Supplementary Figure S3.

Ryanodine, tetracaine, and ATP modulate ER Ca^2+^ release. Ryanodine acts on ryanodine receptors and mediates an agonistic effect at nanomolar–low micromolar concentrations and an antagonistic effect at higher micromolar concentrations (Alexander et al., 2023a). At 10 μM, ryanodine resulted in a time-dependent decrease in CEPIAer FI (control: 77% ± 1.4%; PLNic: 84% ± 3.0%; and PLN-R14del: 75% ± 5.5%). A reduction in force (89% ± 3.1%) and frequency (78% ± 4.1%) observed in the control was not detected either in PLNic or PLN-R14del EHTs (Figure 3C, Supplementary Figure 2A). At 200 μM, ryanodine resulted in a biphasic effect on CEPIAer FI in all three cell lines. An initial decrease after 10 min (control: 68% ± 3.7%; PLNic: 81% ± 3.9%; and PLN-R14del: 84% ± 2.1%) was followed by an increase after 90–210 min (control: 136% ± 5.7%; PLNic: 130% ± 6.0%; and PLN-R14del: 119% ± 2.3%). The initial decrease in CEPIAer FI is compatible with a transient agonistic effect at early time points when the ryanodine concentration at the receptor is building up. In control and PLNic, 200 µM ryanodine had no effect on force and frequency. In contrast, in PLN-R14del, a transient reduction in force and increase in frequency (56% ± 3.4% and 172% ± 9.0%, respectively) were detected (Figure 3D, Supplementary Figure 2B). ATP activates P2Y receptors and mediates a release of ER Ca^2+^ via IP_3_R activation (Reddish et al., 2021). In EHT, ATP (10 mM) led to a time-dependent decrease in CEPIAer FI (control: 70% ± 2.1%; PLNic: 68% ± 4.1%; and PLN-R14del: 76% ± 2.1%). This was accompanied by a discontinuation of contraction (Figure 4A, Supplementary Figure 3A). The latter effect is compatible with the inhibition of L-type Ca^2+^ channels, as previously described for ferret and guinea pig cardiomyocytes, while an opposite effect was described for rats (Vassort, 2001).

**FIGURE 5:**
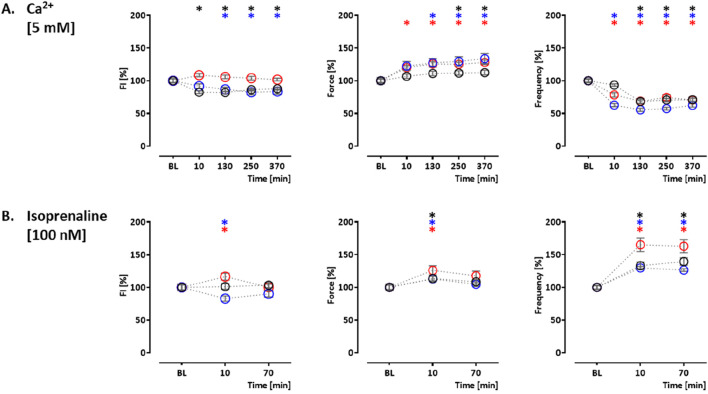
Drug response to calcium (5 mM) and isoprenaline. Effects of calcium (5 mM) and isoprenaline (100 nM) on CEPIAer fluorescence intensity (left), force (middle), and frequency (right) of spontaneously beating hiPSC-CM EHTs from control (O), PLNic (O), and PLN-R14del hiPSC line (O). Data are plotted relative to mean baseline and normalized to TVC per EHT batch. **(A)** Ca^2+^ (5.0 mM; control: n = 25 EHT; TVC = 15 EHT; three batches; PLNic: n = 29 EHT; TVC = 24 EHT; three batches; PLN-R14del: n = 12 EHT; TVC = 9 EHT; one batch). **(B)** Isoprenaline (100.0 nM; control: n = 18 EHT; TVC = 15 EHT; three batches; PLNic: n = 34 EHT; TVC = 28 EHT; three batches; PLN-R14del: n = 28 EHT; TVC = 23 EHT; four batches). One-way ANOVA *versus* baseline with Dunnett’s post-test, *p < 0.05. Mean ± SEM. Asterisk color code indicates the significance for each hiPSC line. The scatter plots of the same data for the different cell lines are shown in Supplementary Figure S4.

Two additional compound effects were studied in the control hiPSC line. The sodium channel blocker tetracaine inhibits ryanodine receptors (Laver and van Helden, 2011) and reduces Ca^2+^ release from the ER. Tetracaine (50 µM) led to an initial increase in CEPIAer FI (122% ± 3.0%). This was partially reverted by an increase in extracellular Ca^2+^ concentration from 1.8 mM to 5 mM. The effects on CEPIAer FI were accompanied by a cessation of contraction due to the inhibition of sodium channels (Grima et al., 1986) (Figure 4B Supplementary Figure 3B). In hiPSC-CM EHTs, this effect has been demonstrated with the sodium channel inhibitors tetrodotoxin, ajmaline, flecainide, quinidine, and mexiletine (Mannhardt et al., 2016). Istaroxime is an activator of SERCA2 and an inhibitor of the Na, K-ATPase (Rocchetti et al., 2005). At 1 and 4 µM, istaroxime led to a non-significant gradual CEPIAer FI increasing trend, which is compatible with the proposed mechanism of action. Force and frequency were not altered at 1 µM, but variability increased at later time points, and at 4 µM, istaroxime led to an abrogation of contractility, which is explained by the Na, K-ATPase inhibition (Figures 4C, D, Supplementary Figure 3B). Similar effects were described by digoxin in hiPSC-CM EHTs (Saleem et al., 2020). Increasing extracellular Ca^2+^ concentration is a well-established experimental method to increase cytosolic Ca^2+^ concentration (Wang et al., 2003), sarcoplasmic reticulum Ca^2+^ loading, and inotropy. Extracellular Ca^2+^ also activates Ca^2+^-sensing receptors and triggers Ca^2+^ release from ER via Gα _q/11_/IP_3_ signaling (Gorkhali et al., 2021). Moreover, the increase in cytosolic calcium triggers the opening of IP_3_R in cardiomyocytes and also ryanodine receptors at higher concentrations (Alexander et al., 2023a; Mak et al., 1998). An increase in extracellular calcium concentration (5 mM) in this EHT model resulted in a decrease in CEPIAer FI in the control and PLNic EHTs (control: 82% ± 2.0%; PLNic: 82% ± 3.8%). In contrast, in PLN-R14del, it had no effect on CEPIAer FI. High extracellular calcium (5 mM) evoked a positive inotropic effect in all three cell lines (control: 113% ± 3.5%; PLNic: 134% ± 7.6%; and PLN-R14del: 128% ± 3.4%), accompanied by a reduction in spontaneous beating frequency (control: 68% ± 2.6%; PLNic: 55% ± 2.4%; and PLN-R14del: 68% ± 4.2%) (Figure 5A, Supplementary Figure 4A).

Isoprenaline-mediated beta-adrenergic activation is a well-established mechanism for PKA-mediated PLN phosphorylation, thereby reducing PLN-mediated SERCA2a inhibition and increasing re-uptake of calcium from the cytosol into the sarcoplasmic reticulum. In addition, PKA phosphorylates IP_3_Rs (Taylor, 2017) and RyRs (Marx et al., 2000), two important components of calcium release from the ER, thereby increasing their open probability. In control and PLNic EHTs, isoprenaline (100 nM) had no effect (control) or a decreasing effect (PLNic: 83% ± 4.0%) on CEPIAer FI. In contrast, isoprenaline induced a transient increase in CEPIAer FI in PLN-R14del (116% ± 5.4%). In all three cell lines, isoprenaline evoked a transient positive inotropic (control: 113% ± 3.7%; PLNic: 113% ± 3.9%; and PLN-R14del: 126% ± 7.0%) and chronotropic effect (control: 140% ± 5.6%; PLNic: 130% ± 2.8%; and PLN-R14del: 165% ± 10.4%; Figure 5B, Supplementary Figure 4B). Supplementary Figure 5 shows the time vehicle controls (TVCs) for these functional analyses. Recordings of CEPIAer FI and force showed stable values with low variability. The beating frequency was less stable and increased in several TVC experiments at late recording time points.

### Cellular CEPIAer fluorescence analysis by FACS and the effect of ER calcium modulators on ER stress marker expression

**FIGURE 6:**
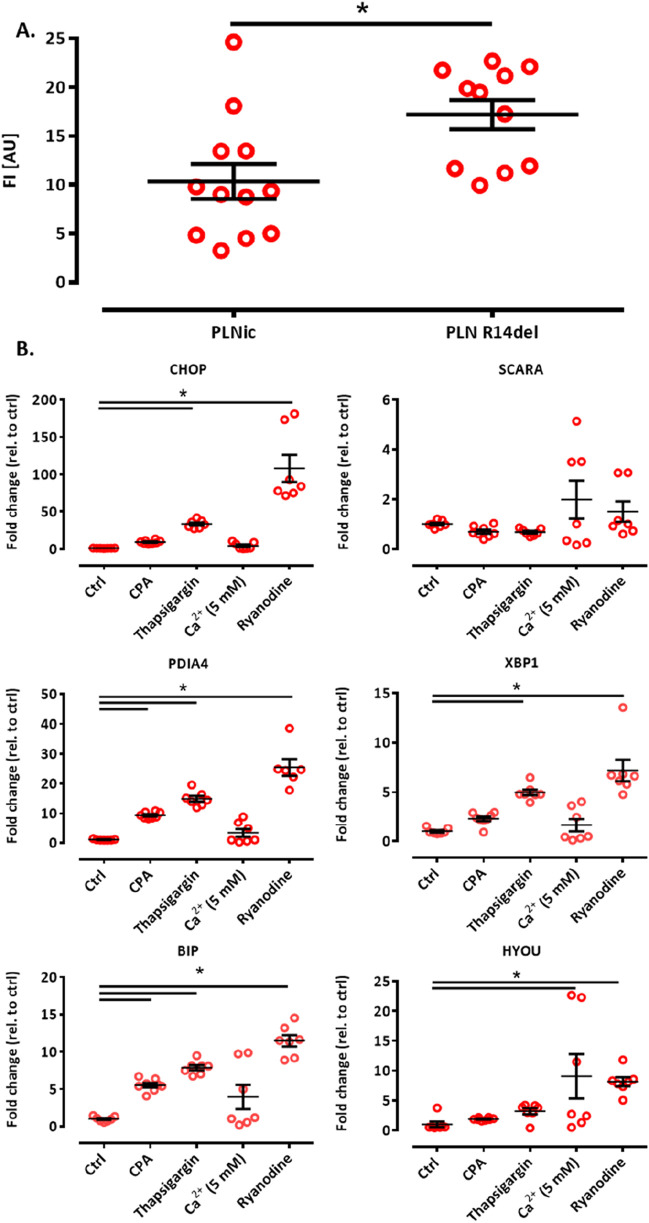
**(A)** ER calcium loading assessment by flow cytometry and ER stress response. Flow cytometry analysis of CEPIAer fluorescence intensity of dissociated hiPSC-CM from PLNic and PLN-R14del EHTs, where each data point represents the average fluorescence intensity of 10,000 individual hiPSC-CM from one EHT. n = 11 PLN-R14del (three batches) and n = 12 PLNic EHTs (three batches). Unpaired Student’s t-test, *p < 0.05. Data are expressed as the mean ± SEM **(B)**. **(A)** Quantitative PCR of ER/UPR stress marker genes in control hiPSC-CM EHT under time/vehicle control (ctrl) or experimental conditions (370 min incubation, cyclopiazonic acid (CPA) 10 μM, thapsigargin 1 μM, calcium 5.0 mM, and ryanodine 10 µM), as indicated. Gene expression was normalized to GUSB, and data were depicted as fold changes relative to control. n = 7–8 EHTs per condition. One-way ANOVA followed by Bonferroni’s post-test for multiple comparisons, *p < 0.05. Data are expressed as the mean ± SEM.

The calcium loading experiment revealed subtle alterations in ER calcium loading in PLN-R14del, which are compatible with higher SERCA-mediated uptake. To investigate whether this finding translates into differences in ER calcium loading at baseline under conditions shown to be associated with cytoplasmic calcium irregularities for PLN-R14del (Cuello et al., 2021), hiPSC-CMs were dissociated from PLNic and PLN-R14del EHTs on day 35–40 of development and subjected to flow cytometry analysis. The analysis revealed higher CEPIAer FI in PLN-R14del (17.2 ± 1.8 AU) than in PLNic (10.3 ± 1.5 AU, mean ± SEM, Figure 6A), suggesting higher ER calcium load in PLN-R14del under baseline conditions. The same CEPIAer AAV batches were used to transduce hiPSC-CMs from both lines. Nevertheless, to account for the variability in CEPIAer transduction and expression, analyses of n = 11–12 EHTs from three different EHT generation batches were performed. Thapsigargin is a known trigger of ER calcium dysregulation and stress response (Abdullahi et al., 2017). To verify this effect in the hiPSC-CM-EHT model and investigate whether such effects were also induced by other modulators of ER calcium homeostasis used in this study, transcript levels of the ER stress response were quantified in control EHTs under the different stress conditions. This validation analysis focused on control hiPSC-CMs as PLN-R14del hiPSC-CMs showed signs of ER stress response at baseline (Cuello et al., 2021). The analysis confirmed an increase in the expression of ER stress markers by thapsigargin and also by other modulators such as CPA, calcium (5 mM), and ryanodine (Figure 6B). These results suggest that the experimental conditions in this study lead not only to changes in the ER calcium load but also to an ER stress response.

## Discussion

The heterozygous PLN-R14del variant causes DCM and severe HF. Previous studies suggest the involvement of ER stress and related pathways such as autophagy (Eijgenraam et al., 2020; Eijgenraam et al., 2021; Te Rijdt et al., 2016; Te Rijdt et al., 2017; Vafiadaki et al., 2024; Cuello et al., 2021; Feyen et al., 2021). In this study, we established the CEPIAer fluorescent sensor in hiPSC-CMs and EHTs. We demonstrate the following findings: 1. perinuclear CEPIAer fluorescence staining and co-localization with ER resident marker in hiPSC-CMs; 2. similar time-dependent effects on CEPIAer FI for EHTs from control, PLNic, and PLN-R14del hiPSC lines in response to indicator compounds for ER calcium uptake and release; 3. unmasking of subtle ER calcium dysregulation in PLN-R14del by calcium loading, indicating higher ER calcium uptake; and 4. higher ER calcium loading in PLN-R14del than in PLNic hiPSC-CM by flow cytometry analysis.

SERCA2 super-inhibition by PLN-R14del was described as a hypothesis for PLN-R14del cardiomyopathy, focusing on SR function (Haghighi et al., 2006; Vafiadaki et al., 2022). This hypothesis was challenged by several studies pointing to just the opposite—reduced SERCA2 inhibition by PLN-R14del. Biochemical analysis of PLN variants reconstituted in lipid membranes revealed reduced protein kinase A-catalyzed phosphorylation of Ser-16 for PLN-R14del and a smaller inhibitory effect on SERCA WT (Hughes and Middleton, 2014). Consistent with this, the PLN-R14del variant was shown to stabilize PLN in its pentameric form and reduce the rate of PLN dissociation from the pentamer, thereby decreasing its ability to regulate SERCA, which is considered to be primarily modulated by monomeric PLN (Cleary et al., 2023). Nuclear magnetic resonance studies provided evidence that PLN-R14del weakens the interactions with the membrane and shifts the conformational equilibrium of PLN toward the disordered R state, which correlates with loss of function (Vostrikov et al., 2015). Studies on the early consequences of PLN-R14del in a transgenic mouse model and hiPSC-CMs revealed evidence of hyper-dynamic calcium handling, shorter half-time of CaT decay, and higher SERCA calcium affinity in PLN-R14del, compatible with diminished SERCA2 inhibition (Maniezzi et al., 2024; Badone et al., 2021).

This study focused on calcium homeostasis in the ER compartment as the subcellular compartment involved in protein biosynthesis, post-translational modification, folding, and degradation of misfolded proteins rather than SR calcium uptake and release during excitation–contraction coupling. Although the separation between ER and SR in cardiomyocytes is not well-understood (Michalak and Opas, 2009), the ER specificity of the CEPIAer-FI in this model is given because its expression is flanked by ER targeting (calreticulin) and retention (KDEL, lysine–aspartic acid–glutamic acid–leucine) sequences (Suzuki et al., 2014). Cytoplasmic calcium loading and isoprenaline treatment revealed subtle differences in PLN-R14del ER calcium loading. Both interventions have effects on several modulators of ER calcium loading. Cytoplasmic calcium loading leads to increased SERCA2-mediated calcium transport and triggers IP_3_R-mediated calcium release (Mak et al., 1998; Alexander et al., 2023b). Although the net effect in both hiPSC control and PLNic lines was a reduction in ER calcium loading, CEPIAer FI remained unchanged in PLN-R14del EHTs, suggesting higher SERCA2 activity. The reduction in ER calcium loading as a summation effect is presumably related to the dominance of ER calcium release mechanisms under these 5 mM calcium loading conditions. The flow cytometry analysis integrated a time period of 35–40 days of development under baseline culture conditions (1.8 mM calcium). This condition was characterized by calcium irregularities in PLN-R14del (Cuello et al., 2021; Feyen et al., 2021). The 1.7-fold higher ER calcium loading in PLN-R14del would be compatible with a dominance of ER calcium uptake mechanisms. The higher ER calcium loading in PLN-R14del could be of biological relevance and contribute to ER stress and ER disease phenotype. The TMCO1 knockout mouse model provides interesting insights in this context. This mouse model lost the ability to reduce ER calcium in response to overload and was characterized by an approximately two-fold increase in ER calcium load, which was associated with a complex and severe phenotype (Wang et al., 2016). The higher ER calcium loading is also well-compatible with the lower abundance of key ER calcium-binding proteins such as calnexin and calreticulin as compensatory mechanisms, as shown by previous proteomics analysis conducted in this model (Cuello et al., 2021). Analysis of CEPIAer FI in intact EHTs would be meaningful and complementary. However, this approach is limited by the high technical variability of the unpaired analysis, particularly with respect to the position of the region-of-interest for the fluorescence signal integration. This technical source of variability leads to large data scatter that might mask potential biological differences.

As an integrated effect of isoprenaline-mediated PKA activation, ER calcium loading was higher in PLN-R14del and was compatible with higher SERCA2 activity and lower PLN-R14del-mediated SERCA2 inhibition, respectively. However, the isoprenaline effect is difficult to classify for two specific reasons. First, the two control lines show different effects: although the unrelated control shows no change in CEPIAer FI, a reduction was observed in PLNic. Second, the EHT cell culture medium did not contain a beta-adrenergic agonist, suggesting that this mechanism contributed little to the higher ER calcium loading detected by flow cytometry analysis. The expected but unobserved effect of SERCA inhibitors on force and frequency is initially difficult to classify. However, it is important to emphasize that similar findings have been described several times using pharmacological SERCA inhibition in different models (Elliott et al., 2012; Chung et al., 2018; Hiranandani et al., 2007) and in a SERCA2 knockout mouse model (Louch et al., 2010). The underlying mechanism of this unexpected observation is not yet understood. In any case, non-linear correlations between SERCA2 activity and force, along with undefined compensatory mechanisms, are likely contributors.

Interestingly, although the effect of cytoplasmic calcium loading and isoprenaline exposure revealed subtle differences in ER calcium loading, a similar effect on SR calcium handling was not apparent, as indicated by a similar inotropic response in the three hiPSC lines examined. This could be related to differences in SERCA2-mediated calcium transport in the ER and SR. SERCA2 exists in two different isoforms, SERCA2a and SERCA2b. The most important structural difference is the replacement of the last four amino acids in SERCA2a by a 49-amino-acid-long tail in SERCA2b. Functionally, the slower transport kinetics and higher calcium affinity of SERCA2b are important. Both isoforms are inhibited by PLN and thapsigargin (Verboomen et al., 1992). In cardiomyocytes, both isoforms SERCA2a and SERCA2b are expressed, with SERCA2a being more abundant (Lipskaia et al., 2014; Dally et al., 2010). The role of SERCA2a in SR calcium regulation is well-established (Eisner et al., 2017). In contrast, SERCA2-mediated calcium transport into the ER in cardiomyocytes is not well-characterized since SERCA2 isoform-specific pharmacological modulators or antibodies do not exist (Michalak and Opas, 2009; Doroudgar and Glembotski, 2013). The relevance of SERCA2b for ER calcium regulation in non-muscle cells and its expression in cardiomyocytes would indicate an important role in ER calcium regulation in cardiomyocytes. Taking these considerations into account, the data from this study would be consistent with less inhibition of PLN-R14del on SERCA2 and, in consequence, a strong cumulative biological effect on ER calcium regulation. Certain limitations of this study have to be considered when interpreting the findings. The relationship between CEPIAer FI, ER calcium loading, and ER functional changes remains incompletely understood, and the downstream consequences on ER pathways were not directly assessed. Although alterations in ER calcium handling are consistent with potential dysregulation of ER function—given the tightly regulated nature of ER calcium homeostasis and its critical role in protein folding, signaling, and induction of cell death (de Ridde et al., 2023; Mekahli et al., 2011)—the findings presented here do not fully explain the complex cardiomyopathy phenotype associated with the PLN-R14del mutation. Instead, they likely represent a contributing mechanism among multiple pathogenic processes. Additionally, the use of hiPSC-CMs, which are known to exhibit an immature phenotype, may limit the translational relevance of the results to adult human cardiomyocytes (Ewoldt et al., 2025). Specifically, the relative contributions of signaling pathways differ and show a dominant role of NCX in EC coupling, with substantial NCX currents and spontaneous activity, while SR function is not fully developed (Seiber et al., 2023; Kim et al., 2015). Detailed studies on how hiPSC-CM immaturity affects ER functions do not exist to date (Ewoldt et al., 2025). Although a rescue experiment targeting SERCA2 activity would be mechanistically informative, the use of inhibitors such as thapsigargin poses significant challenges due to their established role in triggering ER stress and apoptosis with prolonged exposure (Oslowski et al., 2011). Finally, the power and generalizability of the study would have been strengthened by including multiple patient-derived PLN-R14del hiPSC lines to account for inter-individual variability.

## Data Availability

The original contributions presented in the study are included in the article/Supplementary Material further inquiries can be directed to the corresponding author.
