# Supplementary material for: Altered endoplasmic reticulum calcium loading in human PLN-R14del cardiomyopathy
Source: Front Cell Dev Biol. 2025 Jul 28;13:1627985. doi: 10.3389/fcell.2025.1627985 (PMC12336144; doi:10.3389/fcell.2025.1627985)
Supplement: Supplementary file 1 [file DataSheet1.docx]

**Altered endoplasmic reticulum calcium loading in human PLN R14del cardiomyopathy**

**Willem Borbein ^1, 2^*, Lukas Dahmlos ^1, 2^*, Umber Saleem^1, 2^, Marina Reinsch^1, 2^, Ingke Braren^3^, Thomas Schulze^1, 2^, Birgit Klampe^1, 2^, Friederike Cuello^1, 2^, Justus Stenzig^1, 2^, Thomas Eschenhagen^1, 2^, Arne Hansen^1, 2 #^**

^1^University Medical Center Hamburg-Eppendorf, Department of Experimental Pharmacology and Toxicology, 20246 Hamburg, Germany

^2^German Center for Cardiovascular Research (DZHK), Partner site Hamburg/Lübeck/Kiel, Germany

^3^Vector Core Unit, University Medical Center Hamburg-Eppendorf, 20246 Hamburg, Germany

*Joint first author

# Corresponding author

**Supplementary material**

**Supplementary Figure 1**


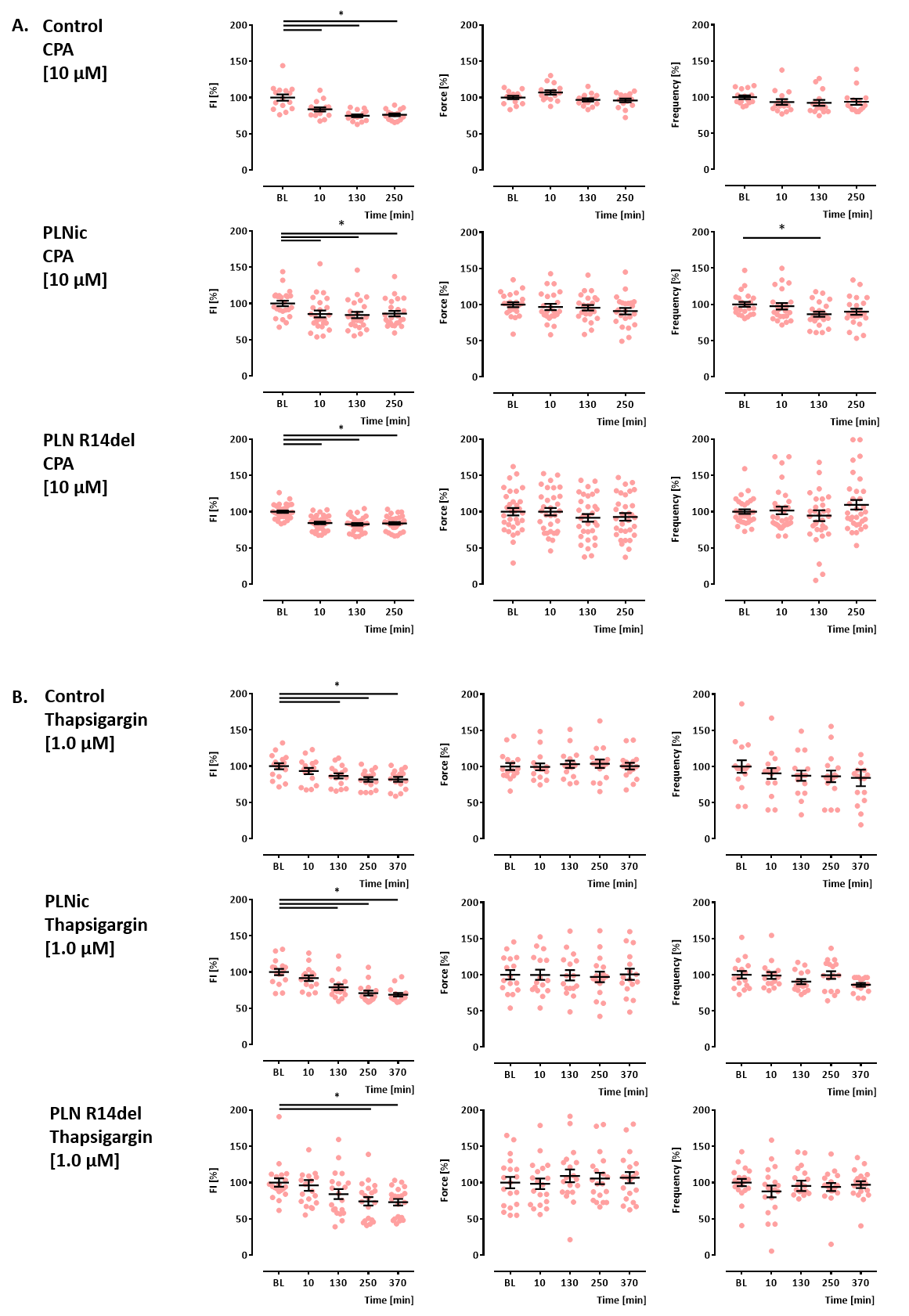


**Supplementary Figure 1.** Drug response to cyclopiazonic acid and thapsigargin. Effects of SERCA2-modulating compound on CEPIAer fluorescence intensity (left), force (middle) and frequency (right) of spontaneously beating hiPSC-CM EHTs from control, PLNic and PLN R14del hiPSC line. Data are plotted relative to mean baseline and normalized to time/vehicle control (TVC) per EHT batch. A: Cyclopiazonic acid (10.0 µM; control: n = 15 EHT; TVC = 14 EHT; 2 batches; PLNic: n = 23 EHT; TVC = 23 EHT; 3 batches; PLN R14del: n = 32 EHT; TVC = 22 EHT; 3 batches). B: Thapsigargin (1.0 µM; control: n = 16 EHT; TVC = 14 EHT; 2 batches; PLNic: n = 16 EHT; TVC = 11 EHT; 2 batches; PLN R14del: n = 19 EHT; TVC = 20 EHT; 3 batches). One-way ANOVA versus baseline with Dunnett`s post-test, * p<0.05. Mean ± SEM.

**Supplementary Figure 2**


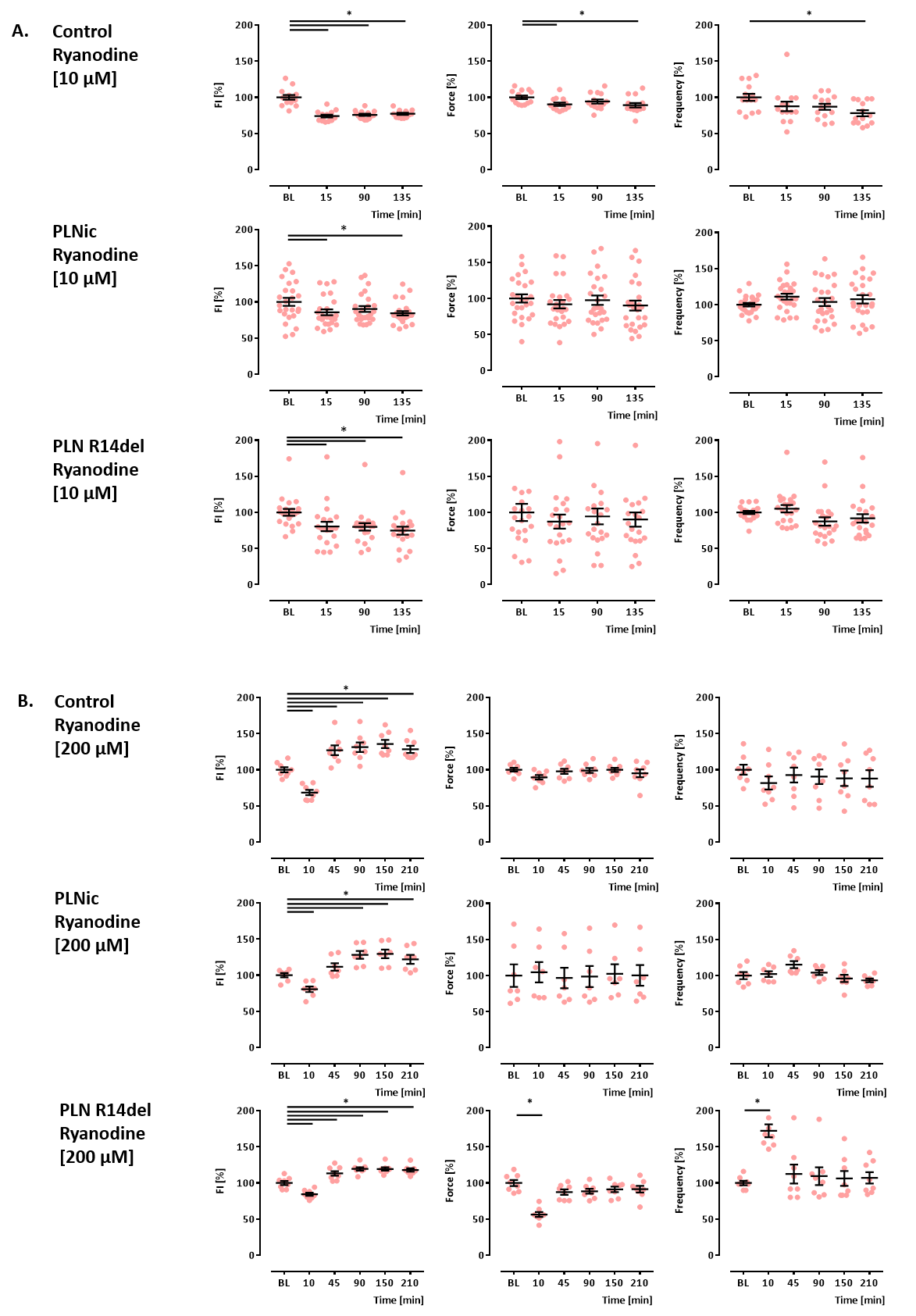


**Supplementary Figure 2.** Drug response to ryanodine. Effects of ryanodine (10.0, 200.0 µM) on CEPIAer fluorescence intensity (left), force (middle) and frequency (right) of spontaneously beating hiPSC-CM EHTs from control, PLNic and PLN R14del hiPSC **lines**. Data are plotted relative to mean baseline and normalized to time/vehicle control (TVC) per EHT batch. A: Ryanodine (10.0 µM; control: n = 14 EHT; TVC = 13 EHT; 2 batches; PLNic: n = 25 EHT; TVC = 20 EHT; 3 batches; PLN R14del: n = 21 EHT; TVC = 19 EHT; 3 batches). B: Ryanodine (200.0 µM; control: n = 8 EHT; TVC = 8 EHT; 2 batches; PLNic: n = 7 EHT; TVC = 8 EHT; 2 batches; PLN R14del: n = 8 EHT; TVC = 8 EHT; 1 batch). One-way ANOVA versus baseline with Dunnett`s post-test, * p<0.05. Mean ± SEM.

**Supplementary Figure 3**


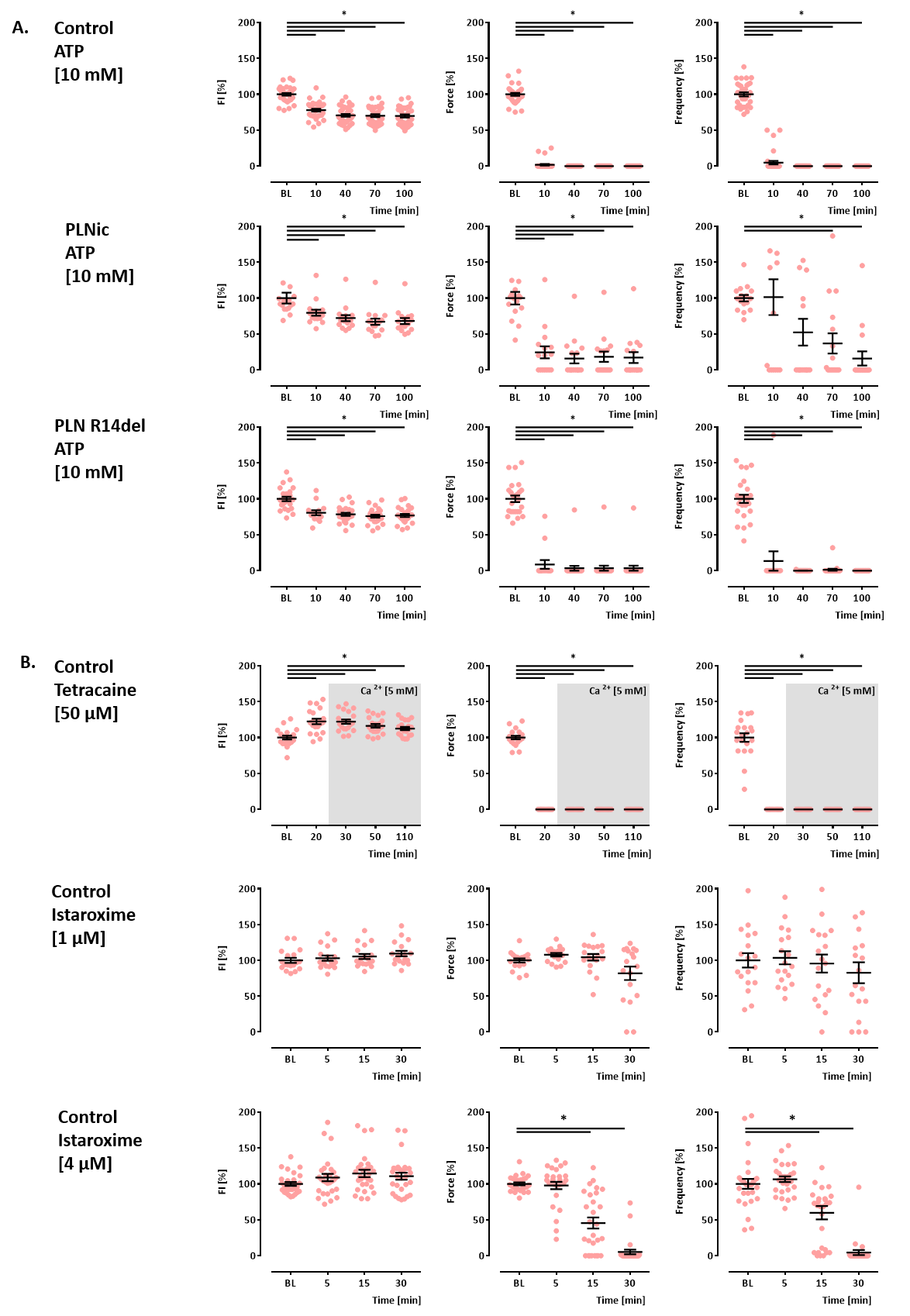


**Supplementary Figure 3.** Drug response to ATP, tetracaine and istaroxime. A: Effects of ATP on CEPIAer fluorescence intensity (left), force (middle) and frequency (right) of spontaneously beating hiPSC-CM EHTs from control, PLNic and PLN R14del hiPSC **lines**. Data are plotted relative to mean baseline and normalized to time/vehicle control (TVC) per EHT batch. ATP (10.0 mM; n = 35 EHT; TVC = 30 EHT; 4 batches; PLNic: n = 16 EHT; TVC = 15 EHT; 2 batches; PLN R14del: n = 25 EHT; TVC = 23 EHT; 3 batches). B. Effects of tetracaine (50 µM) and istaroxime (1, 4 µM) on hiPSC-CM EHTs from control hiPSC line. A: Tetracaine (50.0 µM; n = 20 EHT; TVC = 17 EHT; 2 batches), for the tetracaine experiment extracellular calcium concentration was increased to 5 mM after time point 20 min as indicated in the graphs. B: Istaroxime (1.0 µM; control: n = 18 EHT; TVC = 17 EHT; 3 batches); C: Istaroxime (4.0 µM; n = 28 EHT; TVC = 23 EHT; 4 batches. One-way ANOVA versus baseline with Dunnett`s post-test, * p<0.05. Mean ± SEM.

**Supplementary Figure 4**


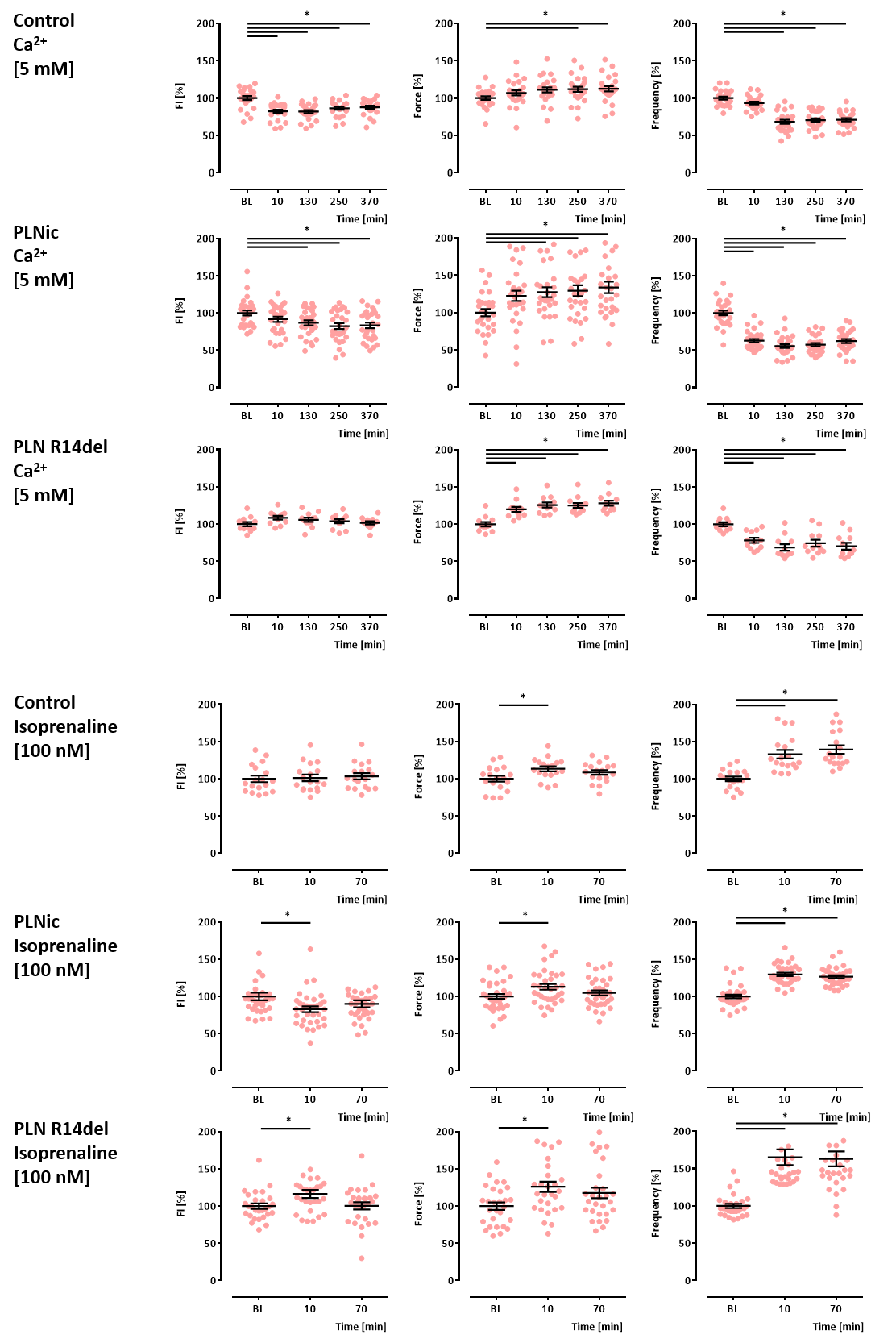


**Supplementary Figure 4.** Drug response to calcium (5 mM) and isoprenaline. Effects of calcium (5 mM) and isoprenaline (100 nM) on CEPIAer fluorescence intensity (left), force (middle) and frequency (right) of spontaneously beating hiPSC-CM EHTs from control, PLNic and PLN R14del hiPSC **lines**. Data are plotted relative to mean baseline and normalized to time/vehicle control (TVC) per EHT batch. A: Calcium (5.0 mM; control: n = 25 EHT; TVC = 15 EHT; 3 batches; PLNic: n = 29 EHT; TVC = 24 EHT; 3 batches; PLN R14del: n = 12 EHT; TVC = 9 EHT; 1 batch). B: Isoprenaline (100.0 nM; control: n = 18 EHT; TVC = 15 EHT; 3 batches; PLNic: n = 34 EHT; TVC = 28 EHT; 3 batches; PLN R14del: n = 28 EHT; TVC = 23 EHT; 4 batches). One-way ANOVA versus baseline with Dunnett`s post-test, * p<0.05. Mean ± SEM.

**Supplementary Figure 5**


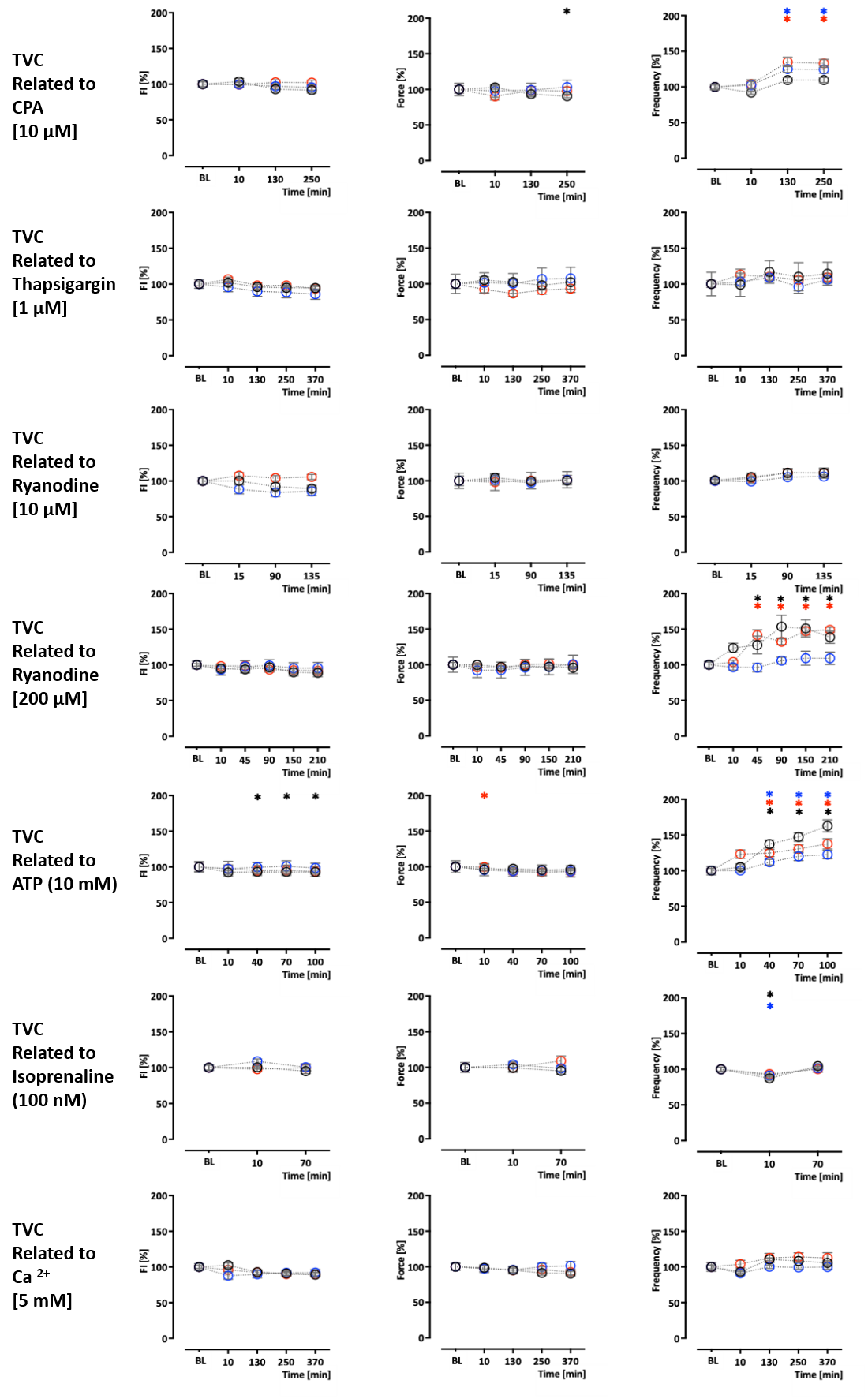

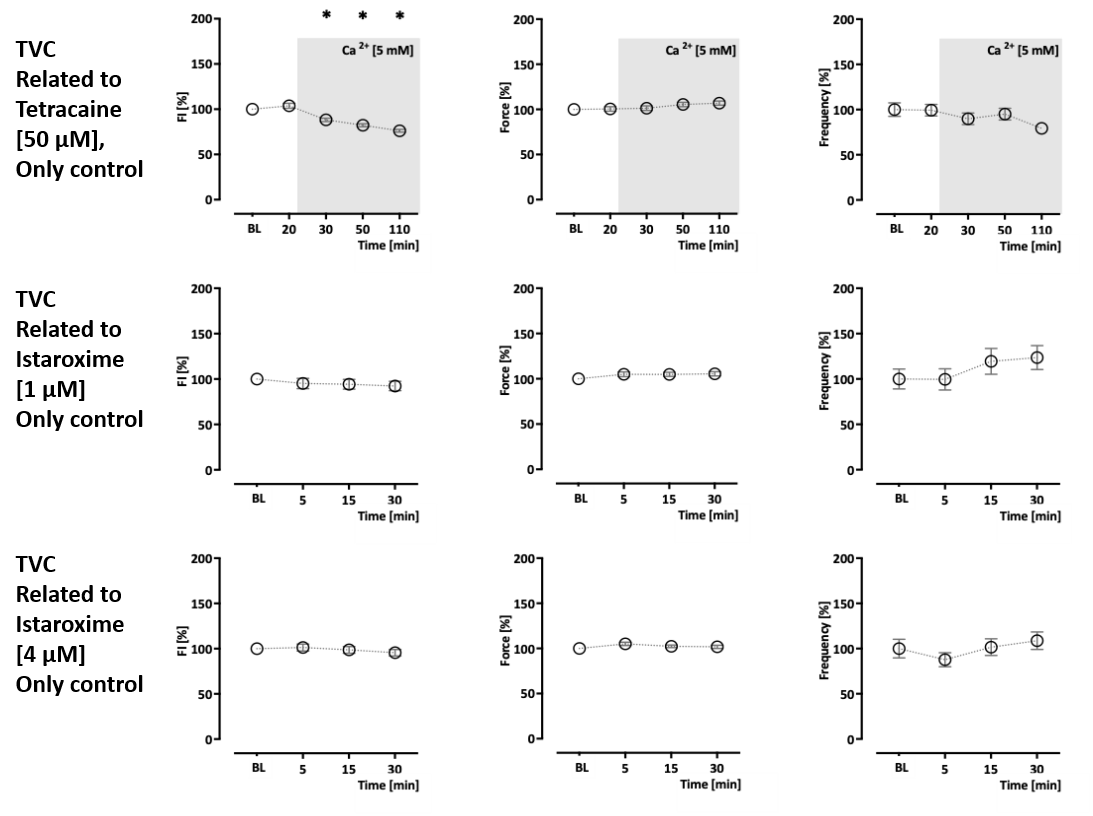


**Supplementary Figure 5**. Time/vehicle control effects. Effect of time and vehicle (time vehicle control, TVC) on CEPIAer fluorescence intensity (left), force (middle) and frequency (right) of spontaneously beating hiPSC-CM EHTs from control (**O**), PLNic (**O**) and PLN R14del (**O**) hiPSC **lines**. Data are plotted relative to mean baseline per EHT batch. Replicate numbers of TVC: CPA experiment: control: n = 14 EHT; 2 batches; PLNic: n = 23 EHT; 3 batches; PLN R14del: n = 22 EHT; 3 batches; thapsigargin experiment: control: n = 14 EHT; 2 batches; PLNic: n = 11 EHT; 2 batches; PLN R14del: n = 20 EHT; 3 batches; ryanodine [10 µM] experiment: control: n = 13 EHT; 2 batches; PLNic: n = 20 EHT; 3 batches; PLN R14del: n = 19 EHT; 3 batches; ryanodine [200 µM] experiment: control: n = 8 EHT; 2 batches; PLNic: n = 8 EHT; 2 batches; PLN R14del: n = 8 EHT; 1 batch; ATP experiment: control: n = 30 EHT; 4 batches; PLNic: n = 15 EHT; 2 batches; PLN R14del: n = 23 EHT; 3 batches; isoprenaline experiment: control: n = 15 EHT; 3 batches; PLNic: n = 28 EHT; 3 batches; PLN R14del: n = 23 EHT; 4 batches; calcium experiment: control: n = 15 EHT; 3 batches; PLNic: n = 24 EHT; 3 batches; PLN R14del: n = 9 EHT; 1 batch; tetracaine experiment: control: n = 17 EHT; 2 batches, for the tetracain experiment extracellular calcium concentration was increased to 5 mM after time point 20 min as indicated in the graphs; istaroxime [1 µM] experiment: control: n = 17 EHT; 3 batches; istaroxime [4 µM] experiment: control: n = 23 EHT; 4 batches; data are plotted relative to mean baseline per EHT batch. For tetracain experiment extracellular calcium concentration was increased to 5 mM after time point 20 min as indicated in the graphs. One-way ANOVA versus baseline with Dunnett`s post-test, * p<0.05. Mean ± SEM. Color code indicate significance for control (*), PLNic (*) and PLN R14del (*).
